# Mucin O-glycan degradation by GH101 underpins mucosal persistence of *Bifidobacterium bifidum* PRL2010

**DOI:** 10.1007/s00253-026-13964-1

**Published:** 2026-07-18

**Authors:** Aryanna Muscò, Giulia Longhi, Emanuele Selleri, Emma Gennaioli, Gabriele Andrea Lugli, Chiara Tarracchini, Massimiliano Giovanni Bianchi, Ovidio Bussolati, Marco Ventura, Francesca Turroni

**Affiliations:** 1 Laboratory of Probiogenomics, Department of Chemistry, Life Sciences, and Environmental Sustainability, University of Parma, Parma, Italy; 2 Microbiome Research Hub, University of Parma, Parma, Italy; 3 Laboratory of General Pathology, Department of Medicine and Surgery, University of Parma, Parma, Italy

**Keywords:** Bifidobacteria, Mucin metabolism, Human gut microbiota, Host-microbe interaction, Glycosyl hydrolases

## Abstract

**Abstract:**

Host-derived mucin O-glycans constitute a key chemical component of the human intestinal niche and are continuously encountered by gut-adapted bifidobacterial cells, thereby playing a pivotal role in host–microbe interactions. Among these microbes, *Bifidobacterium bifidum* is one of the most persistent members of the human gut microbiota. Comparative genomic analyses of mucosa-associated strains of this species have revealed a gene conserved across its pangenome, predicted to encode a GH101 glycoside hydrolase involved in mucin degradation. In this study, we performed a molecular characterization of the GH101 enzyme encoded by *B. bifidum* PRL2010. Insertional mutagenesis of the GH101 gene from the PRL2010 genome resulted in a marked reduction in bacterial adhesion to mucin-secreting epithelial cells, along with a significant growth difference when N-acetyl-galactosamine was provided as the sole carbon source. These findings indicate that GH101 mediates the initial cleavage of O-GalNAc core 1 structures, representing a crucial step for the adhesion to human mucosa. However, this enzyme represents only one component of the broader set of glycosidases required for the complete degradation of host mucins. Overall, our results establish a mechanistic link between metabolic specialization and ecological fitness, providing insights into how mucin-adapted commensals may contribute to interactions with the human gut mucosa.

**Key points:**

• *The GH101 glycoside hydrolase of Bifidobacterium bifidum PRL2010 plays a pivotal role in host mucin utilization by initiating the cleavage of O-GalNAc core 1 structures.*

• *Insertional mutant of the GH101 gene impairs both mucosal adhesion and growth on N-acetyl-galactosamine.*

• *These findings reveal a mechanistic connection between mucin glycan metabolism and ecological fitness by mucin-adapted bifidobacterial commensals.*

**Graphical Abstract:**

Within the intestinal ecosystem, *Bifidobacterium bifidum* PRL2010 utilizes epithelial mucin through the activity of glycoside hydrolase (GH) enzymes, releasing sugars that support microbial cross-feeding. GH101-deficient mutants lose mucin-degrading capacity and show impaired growth on mucin, demonstrating the essential role of GH101 in mucin utilization and gut microbe interactions. Created in BioRender. Turroni, F. (2026) https://BioRender.com/jhll48f

**Figure:**
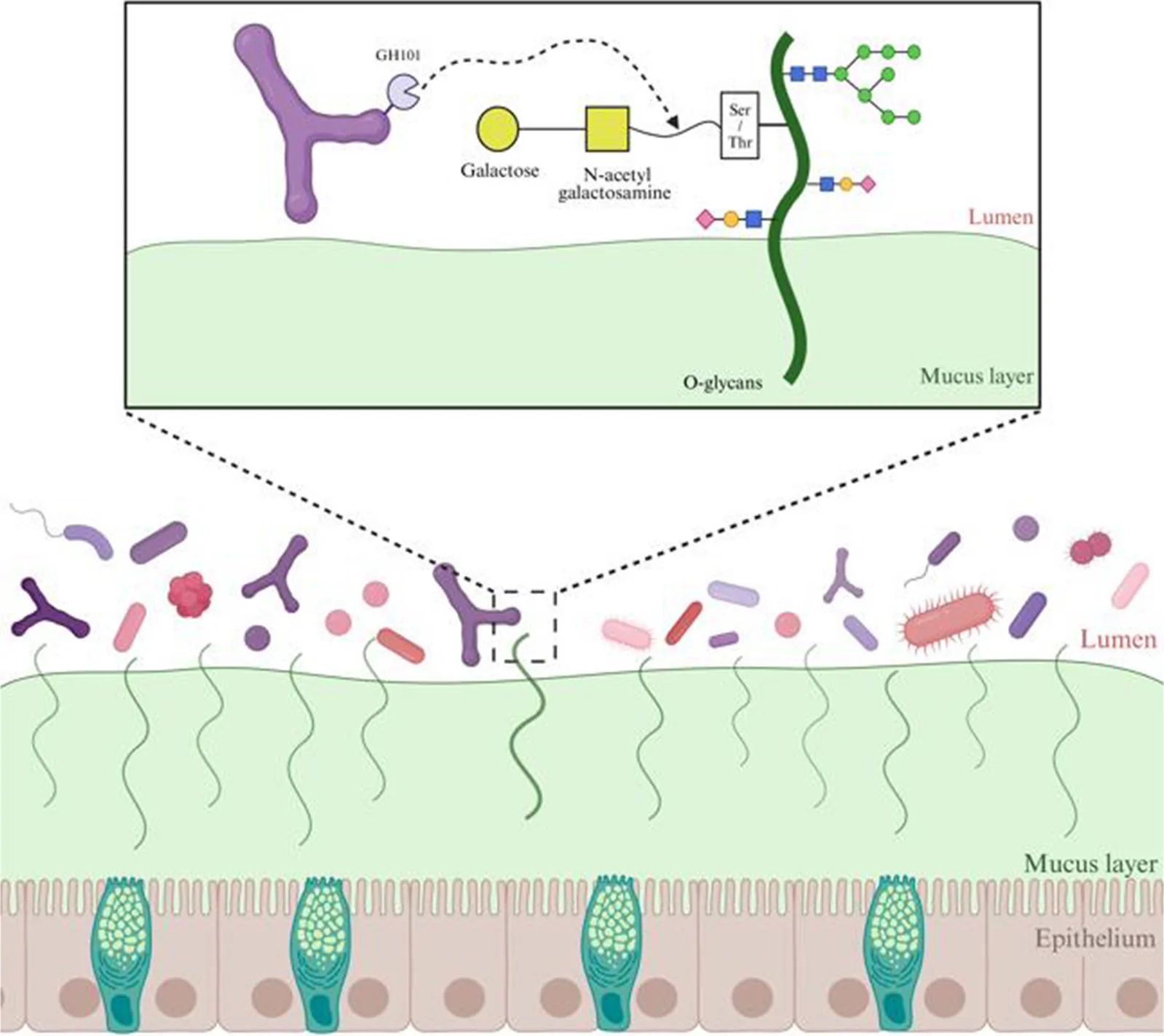

**Supplementary Information:**

The online version contains supplementary material available at https://doi.org/10.1007/s00253-026-13964-1.

## Introduction

Bifidobacteria are prominent commensal members of the human gastrointestinal microbiota and play an important role in host health. Their beneficial physiological effects have made *Bifidobacterium* species a major focus of microbiological and biomedical research (Koropatkin et al. 2012; O’Callaghan & van Sinderen 2016; Turroni et al. 2014). Early colonization by bifidobacteria is pivotal in shaping the infant gut microbiota, supporting immune development, mucosal barrier integrity, and resistance to pathogenic invasion (Kumar et al. 2020; Samarra et al. 2025). *Bifidobacterium bifidum* PRL2010 is an early colonizing human gut commensal, originally isolated from the stool of a 3-month-old infant, and is widely recognized as a model organism for studying host glycan foraging by bifidobacteria (Turroni et al. 2010a, b). PRL2010 strain possesses a genome enriched in carbohydrate-active enzymes, carbohydrate-binding modules, and dedicated transporters tailored for the metabolism of host-derived glycans, including mucin O-GalNAc glycans and human milk oligosaccharides (HMOs) (Katoh et al. 2017; Milani et al. 2015; Turroni et al. 2011a, b). Previous transcriptomic studies have demonstrated the involvement of GH101 in host glycan metabolism, showing increased expression of the GH101-encoding gene when PRL2010 was grown on mucin (Tarracchini et al. 2023). These findings suggest a functional role for GH101 in the degradation of complex host glycans within the intestinal mucosal niche (Turroni et al. 2010a, b), although the underlying mechanisms remain to be elucidated.

Mucin O-glycans (O-GalNAc glycans) are covalently α-linked via an N-acetylgalactosamine (GalNAc) moiety to the hydroxyl group of serine or threonine residues through an O-glycosidic bond. These heavily glycosylated proteins form the protective mucosal layer of the intestine and serve both as a nutrient source and as a physical structure that commensal bacteria must colonize. *B. bifidum* PRL2010 is a highly mucosa-adapted member of the human gut microbiota, characterized by enhanced persistence within the intestinal ecosystem and stable host association, including sex-biased colonization patterns (Tarracchini et al. 2023). The glycoside hydrolase family GH101 was first characterized in *Bifidobacterium longum* (Fujita et al. 2005). GH101 enzymes adopt a (β/α)\₈-barrel fold, with Asp and Glu residues forming the catalytic core (Suzuki et al. 2009). They specifically target core 1 O-linked glycans (Gal-β1,3-GalNAc-α-R), initiating their degradation through the action of endo-α-N-acetylgalactosaminidases such as EngBF from *Bifidobacterium longum* (EC 3.2.1.97), which cleaves mucin-type O-glycans by releasing the disaccharide Galβ1,3-GalNAc. This process contributes to the generation of simpler molecules that can be metabolized by the bacterium itself and by other microorganisms (Gregg et al. 2015).

Comparative genomic analyses of mucosa-associated *B. bifidum* strains have identified a conserved set of genes associated with persistence and host adaptation (Fujita et al. 2005; Naumoff 2010; Turroni et al. 2010a, b; Tarracchini et al. 2023). Among these, genes encoding GH101 are conserved across *B. bifidum* genomes (Glover et al. 2022) but are largely absent in other bifidobacteria (Li et al. 2024), suggesting a species-specific adaptation that enhances mucin degradation and mucosal persistence.

Together with other glycoside hydrolases, such as GH129, GH33, and GH29, GH101 contributes to the release of monosaccharides, including GalNAc, Gal, and GlcNAc, thereby supporting both metabolic activity and colonization of the intestinal mucosa (Fujita et al. 2005; Milani et al. 2015; Takada et al. 2023). Although the biochemical activity of GH101 has been characterized in other bacteria (Gregg et al. 2015; Zhou et al. 2025), its functional role in *B. bifidum* PRL2010 remains unclear. Given that mucin degradation underpins not only nutrient acquisition but also epithelial adhesion, biofilm formation, and microbial competition, we suggest that GH101 is a key determinant of both mucin utilization and host interactions. To test this hypothesis, we constructed a GH101-deficient mutant of *B. bifidum* PRL2010 (GH101::pFREM30) and assessed its growth on host-derived glycans, as well as its adhesion to mucin-secreting HT29-MTX intestinal epithelial cells.

## Materials and methods

### Comparative genomics analysis and prediction of GH101-like orthologous genes

Complete and partial genome sequences of 117 *Bifidobacterium* (sub)species were retrieved from the RefSeq NCBI database, resulting in 2665 high-quality genomes (O’Leary et al. 2016) (Supplementary Table S1). The curated genomic sequences were annotated using PROKKA (Seemann 2014), and type strain gene annotations were used for the pangenome, as well as for the phylogenetic analysis performed by Roary (Page et al. 2015) (identity threshold of 80% for the formation of clusters of orthologous genes, COGs) and RAxML (Stamatakis 2014). The ability to use different carbon sources was evaluated using the dbcan3 software (Zheng et al. 2023). The multialignment and identity matrix of the sequences exhibiting GH101-like domains were generated using MAFFT v7.505 (Katoh et al. 2002).

### Sequence similarity analysis

Protein sequence similarity between *B. longum* EngBF (AAX44931.1) and BBPR_RS01340 was evaluated using BLASTP (NCBI; https://blast.ncbi.nlm.nih.gov/Blast.cgi) with default settings. Amino acid identity and sequence coverage were calculated based on the best-scoring alignment.

### Bacterial strains and cultivation conditions

For plasmid transformation procedures, Escherichia coli EC101 was selected as the recipient strain according to the method described by Law et al. (1995). Cultures were propagated at 37 °C in Luria–Bertani (LB) broth (Scharlab, Spain), with the addition of chloramphenicol (CHL) to a final concentration of 25 µg/mL in order to maintain selective growth conditions. *B. bifidum* PRL2010 wild-type and *B. bifidum* GH101::pFREM30 strains were routinely cultivated under anaerobic conditions (Concept 400, Ruskinn) at 37 °C in a modified de Man-Rogosa-Sharpe (mMRS) broth, without glucose and supplemented with 2% lactose and 0.05% (w/v) L-cysteine hydrochloride. *B. bifidum* GH101::pFREM30 was cultivated in the presence of CHL at a concentration of 5 µg/mL to assess plasmid stability (Rizzo et al. 2024). For experimental assays, the bifidobacteria mutant strain was grown at the lowest effective antibiotic concentration, and cells were harvested, washed, and resuspended in the appropriate basal medium before inoculation at an OD₆₀₀ ≈ of 0.1. The *B. bifidum* PRL2010 strain employed in this study was originally isolated from the fecal sample of a breastfed infant (Turroni et al. 2010a, b) and is deposited in the LMG culture collection with the code LMG S-28692.

### Plasmid construction

Transformants were selected by plating the manipulated cells on LB agar supplemented with 25 μg/mL chloramphenicol, and colonies were subsequently screened by colony PCR. Plasmids were constructed as previously described (Rizzo et al. 2024). Briefly, pFREM30 was assembled using the Golden Gate cloning approach (Engler & Marillonnet 2014). The chloramphenicol resistance gene (CmR) was amplified from the pFREM28 backbone using the Type IIS enzyme SapI, transformed into *E. coli* EC101, and subsequently confirmed by PCR and sequencing (Hoedt et al. 2021). For the construction of plasmid pFREM30-01340, an internal fragment of the BBPR_RS01340 gene from *B. bifidum* PRL2010 chromosomal DNA was amplified by PCR employing BBPR_RS01340 fw (5′**-** aataatgtgcacAGAAGCGCACCTACACCG −3′) and BBPR_RS01340 rv (5′- GTAGGCCTGATGCCGCTGgagctctaataa −3′) primers, and the amplicon is 296 bp. The amplicon and pFREM30 plasmid were digested with *Xho*I and *Apa*LI, ligated using the T4 DNA ligase, and introduced into *E. coli* EC101. The integration of pFREM30-01340 into an internal region of BBPR_RS01340 via homologous recombination disrupts the gene structure, abolishes the continuity of the coding sequence, and results in a truncated, non-functional protein. Therefore, insertional inactivation of BBPR_RS01340 is unlikely to exert a polar effect on downstream genes because it is not part of a polycistronic operon.

The integrity of the plasmid is verified with the amplification with specific primers: MCS1 fw (5′-CAA TCG CCA ACG AAT CGC CAA C −3′) and MCS2 rv (5′- CGT CCC GTC TCC TAT AAC CCT C −3′).

### Electroporation and PRL2010 mutant construction

As previously reported, *B. bifidum* is highly resistant to genetic transformation due to its thick cell wall and active restriction–modification (R/M) systems (Serafini et al. 2012). To overcome this, an optimized high-efficiency transformation protocol was developed. Methylome analysis using PacBio sequencing and REBASE predictions identified multiple Type I and Type II R/M recognition motifs in *B. bifidum* PRL2010 (Rizzo et al. 2024). These motifs were removed from the plasmids pNZ003 and pFREM30 to prevent degradation of unmethylated foreign DNA.

An overnight culture of *B. bifidum* PRL2010 was inoculated into fresh mMRS broth supplemented with 7% (v/w) sucrose and grown at 37 °C until reaching the exponential phase (OD₆₀₀ 0.5–0.6). Cells were then harvested by centrifugation at 4500 × *g* for 10 min at 4 °C, washed twice with ice-cold citrate–sucrose buffer (0.5 M sucrose, pH 5.8), and finally resuspended in 250 μL of the same buffer. 100 μL of PRL2010 competent cells were combined with 1.5 μg of plasmid in a precooled disposable electroporation cuvette with a 0.2 cm interelectrode gap (Cell Project, Kent, UK). Electroporation was carried out using a Gene Pulser apparatus (Bio-Rad, UK) applying the following parameters: 2.5 kV, 25 μF, and 200 Ω. Immediately after the pulse, bacterial cells were recovered in 950 μL of sMRS medium and incubated anaerobically at 37 °C for 3 h. The recovered cultures were subsequently spread onto mMRS agar plates supplemented with chloramphenicol at a concentration of 5 μg/mL and incubated under anaerobic conditions at 37 °C for 48 h.

Putative mutant colonies were screened by colony PCR employing the primers GH101_fw (5′-GTACTACCACCGCGTCG-3′) and GH101_rv (5′-CCATCAGGGTGTTCATGTC-3′), targeting chromosomal regions flanking the gene of interest, together with the primer CLMC_022 (5′**-** CGAATCGCCAACGTTTTC −3′), designed to anneal to the integrated pFREM30 plasmid sequence. The expected PCR products had approximate sizes of 2382 bp and 518 bp, respectively.

### Mutant stability

The stability of the integrated pFREM30 construct was assessed by spot assays and further validated by colony PCR. *B. bifidum* GH101::pFREM30 cultures were grown in mMRS medium either in the presence of chloramphenicol (5 μg/mL) or in antibiotic-free conditions. For 14 consecutive days, cultures were subcultured daily into fresh medium while preserving the corresponding selective or non-selective environment. Every 48 h, overnight cultures obtained from each condition were serially diluted to 10⁻^4^, 10⁻^5^, and 10⁻⁶, and aliquots of each dilution were spotted onto mMRS agar plates with or without chloramphenicol (5 μg/mL). After incubation under anaerobic conditions at 37 °C for 48 h, a single colony from each biological replicate was randomly selected for colony PCR analysis using primers GH101_fw and CLMC_022 to confirm maintenance of the integrated construct. All experiments were performed in triplicate.

### Plasmid maintenance assay under sub-selective conditions

The mutant strain was cultured overnight under anaerobic conditions at 37 °C in mMRS broth lacking glucose and supplemented with 2% lactose, 0.05% (w/v) L-cysteine hydrochloride, and CHL at a final concentration of 5 µg/mL. Subsequently, the culture was adjusted to an OD₆₀₀ of approximately 1 in the same medium without antibiotic and used to inoculate tubes with a final OD₆₀₀ approximately 0.1 containing mMRS broth (without glucose, supplemented with 2% lactose and 0.05% (w/v) L-cysteine hydrochloride) and decreasing concentrations of antibiotic ranging from 5 µg/mL to 0.1 µg/mL, in a final volume of 1.5 mL. Cultures were inoculated in triplicate and incubated anaerobically at 37 °C for 24 h. Following growth, plasmid retention was assessed by extracting bacterial DNA using a standard rapid glass bead-based protocol (Tarracchini et al. 2025). Briefly, 1 mL of culture was centrifuged at 6000 rpm for 5 min, washed, and resuspended in 1 mL of distilled water. The cell suspension was then mixed with approximately 250 mg of glass beads and homogenized using a bead-beater for 2 min. To improve DNA yield, samples were incubated on ice for 2 min, and the cycle was repeated twice. Samples were subsequently centrifuged at 10,000 rpm for 2 min, and the supernatant was collected and quantified by spectrophotometric analysis (Tarracchini et al. 2025). The extracted DNA was then subjected to PCR amplification using plasmid-specific primers (GH101_fw and CLMC_022), and the resulting amplicons were analyzed by agarose gel electrophoresis (1.5% agarose) using a 1 kb DNA ladder.

### Growth assay in the presence of mucin as a unique carbon source

To investigate potential differences in mucin metabolism, both the wild-type strain *B. bifidum* PRL2010 and the mutant *B. bifidum* GH101::pFREM30 were first grown overnight until they reached the exponential phase (OD600 ~ 0.8–1.0). Based on the growth curve assessment for each strain, cells were then collected by harvesting and resuspended in carbon-free mMRS medium to a standardized OD600 of 1. Subsequently, cultures were further diluted to an OD600 of 0.1 in a final volume of 270 µL of mMRS supplemented with mucin as the only available carbon source. The mucin solution had been previously sterilized through filtration using a 0.2 µm membrane filter and was added to autoclaved mMRS medium lacking any carbon source (121 °C for 15 min). The prepared samples were distributed into 96-well microtiter plates and incubated under anaerobic conditions. Growth kinetics were monitored at 37 °C by recording OD600 values every 30 min using a Stratus microplate reader (Cerillo, Charlottesville, VA, USA), while continuous agitation at 250 rpm was ensured by a Microplate Shaker PMS-1000i (Grant Instruments, UK). Since the shaking system cannot be paused or programmed to stop before measurements, all readings were performed in continuous kinetic mode. All experimental conditions were carried out in biological triplicate.

### Evaluation of bacterial viability assay

For the assessment of bacterial viability, both the wild-type *B. bifidum* PRL2010 and the engineered strain *B. bifidum* GH101::pFREM30 were initially cultured overnight until they reached the exponential growth phase (OD600 approximately 0.8–1.0). Cells were then collected and washed twice using sterile phosphate-buffered saline (PBS) (Sigma, Germany). Following the washing steps, bacterial pellets were resuspended in 20 mL of either mMRS medium or mMRS supplemented with 0.5% porcine gastric mucin (Type III, Sigma, Germany), which served as the sole carbon source. The starting inoculum was standardized to an OD600 of about 0.1. Each condition was prepared in three independent biological replicates and incubated anaerobically at 37 °C for 48 h. At specific time intervals (0, 6, 12, 24, 36, and 48 h), culture aliquots were withdrawn and used for viability measurements. In parallel, samples were plated onto mMRS agar and incubated under anaerobic conditions at 37 °C for 48 h. Viable cell numbers were determined by counting colony-forming units (CFU).

### HT29-MTX cells adhesion assay

Adhesion experiments were carried out following the protocols previously reported by Guglielmetti et al. (2008) and Serafini et al. (2012). HT29-MTX cells were maintained in Dulbecco’s modified Eagle’s medium (DMEM) supplemented with 10% (v/v) fetal bovine serum (FBS), 10 mM HEPES, 4 mM L-glutamine, 100 µg/mL streptomycin, and 100 U/mL penicillin, under standard culture conditions. For the assays, epithelial cells were seeded into 3-cm^2^ Petri dishes and allowed to reach confluence, corresponding to approximately 3 × 10^5^ cells per dish. The bacterial strains *B. bifidum* PRL2010, GH101::pFREM30, and *B. bifidum* subsp. *animalis* Bb-12 were prepared as previously described and adjusted to a concentration of about 1 × 10⁸ CFU/mL. Bacterial suspensions were then centrifuged at 3000 rpm for 8 min, washed three times, and finally resuspended in 1 mL of sterile phosphate-buffered saline (PBS, pH 7.4).

The bacterial cells were incubated with HT29-MTX monolayers for 1 h at 37 °C to allow adhesion. After incubation, non-adherent bacteria were removed by two gentle PBS washes. The monolayers were then fixed in methanol for 8 min and stained with Giemsa solution (1:20; Sigma-Aldrich, Milan, Italy) for 30 min in the dark.

After staining, coverslips were washed, mounted, and observed using a Zeiss Axiovert 200 phase-contrast microscope at 100× magnification (oil immersion, 1.4 NA). For each strain, adherent bacteria were quantified by counting nine randomly selected microscopic fields. The adhesion index was calculated as the average number of bacterial cells attached per 100 HT29-MTX cells (Guglielmetti et al. 2008; Rizzo et al. 2023). Statistical analysis was performed using Welch’s *t*-test, and all experiments were conducted in biological triplicate.

### Carbohydrate growth assay

*B. bifidum* PRL2010 and *B. bifidum* PRL2010 GH101::pFREM30 were grown overnight in mMRS medium at 37 °C under anaerobic conditions. Cells were subsequently harvested and resuspended in mMRS medium lacking a carbon source to an optical density (OD600) of 1. The cultures were further diluted to an OD600 of 0.1 in 270 µL of mMRS medium supplemented with a specific carbon source, then dispensed into a 96-well microtiter plate and incubated in an anaerobic cabinet. Each carbohydrate solution was filter-sterilized using a 0.2 µm membrane filter prior to use, then added to autoclaved mMRS medium lacking a carbon source. Bacterial growth was monitored by measuring OD600 every 30 min at 37 °C under anaerobic conditions using a Stratus microplate reader (Cerillo, Charlottesville, VA, USA), with continuous shaking at 160 rpm provided by a Microplate Shaker PMS-1000i (Grant Instruments, Shepreth, Cambridgeshire, UK). All experiments were performed in kinetic mode with continuous shaking, as the shaker is not programmable and cannot be paused prior to OD measurement. Cultures were grown in triplicate. The carbohydrates tested in this study were purchased from Merck (Germany) and included lactose, N-acetyl-D-glucosamine (GlcNAc), and N-acetyl-D-galactosamine (GalNAc).

### RNA extraction and bacterial gene expression analysis qRT-PCR

Total RNA was extracted following previously established protocols (Turroni et al. 2010a, b). In brief, cell pellets were resuspended in 1 mL of QIAZOL reagent (Qiagen, United Kingdom; Cat. no.: 79306) and combined with 0.8 g of 10^6^ μm glass beads (Sigma). Cell disruption was achieved using a bead-beating procedure consisting of alternating 2-min shaking cycles and 2-min incubations on ice, repeated multiple times. Lysates were centrifuged at 12,000 rpm for 15 min, and RNA was recovered from the aqueous phase. RNA purification was performed using the RNeasy Mini Kit (Qiagen, Germany), in accordance with the manufacturer’s instructions. RNA integrity was assessed via TapeStation 2200 (Agilent Technologies, USA), while RNA load and purity were determined spectrophotometrically (Eppendorf, Germany).

To validate *perB* overexpression in wild-type and mutant strains, we performed quantitative reverse transcription PCR (qRT-PCR) assays targeting the genes of interest. Reverse transcription of RNA into cDNA was carried out using the iScript Select cDNA synthesis kit (Bio-Rad Laboratories) under the following thermal protocol: 5 min at 25 °C, 30 min at 42 °C, and 5 min at 85 °C. Quantitative real-time PCR (qRT-PCR) was performed using SYBR Green technology with PowerUp SYBR Green Master Mix (Thermo Fisher, US) on a Bio-Rad CFX96 detection system, following the manufacturer’s instructions (Bio-Rad). Primers specific to each gene were designed and validated in silico using Primer-BLAST (Supplementary Table S2). The qRT-PCR was performed with the following cycling parameters: an initial hold at 50 °C for 2 min, followed by 95 °C for 2 min, and 40 amplification cycles of 95 °C for 15 s and 60 °C for 1 min. Gene expression levels were normalized to housekeeping genes *atpD*, *rpoB,* and *LDH* (Turroni et al. 2011a, b). For each reaction, 15 ng of cDNA template was used. Negative controls (no DNA) were included for each primer pair in every run. Standard curves were generated using the Bio-Rad CFX96 software to assess amplification efficiency.

## Results

### Distribution of the GH101 family among bifidobacteria

**Fig. 1:**
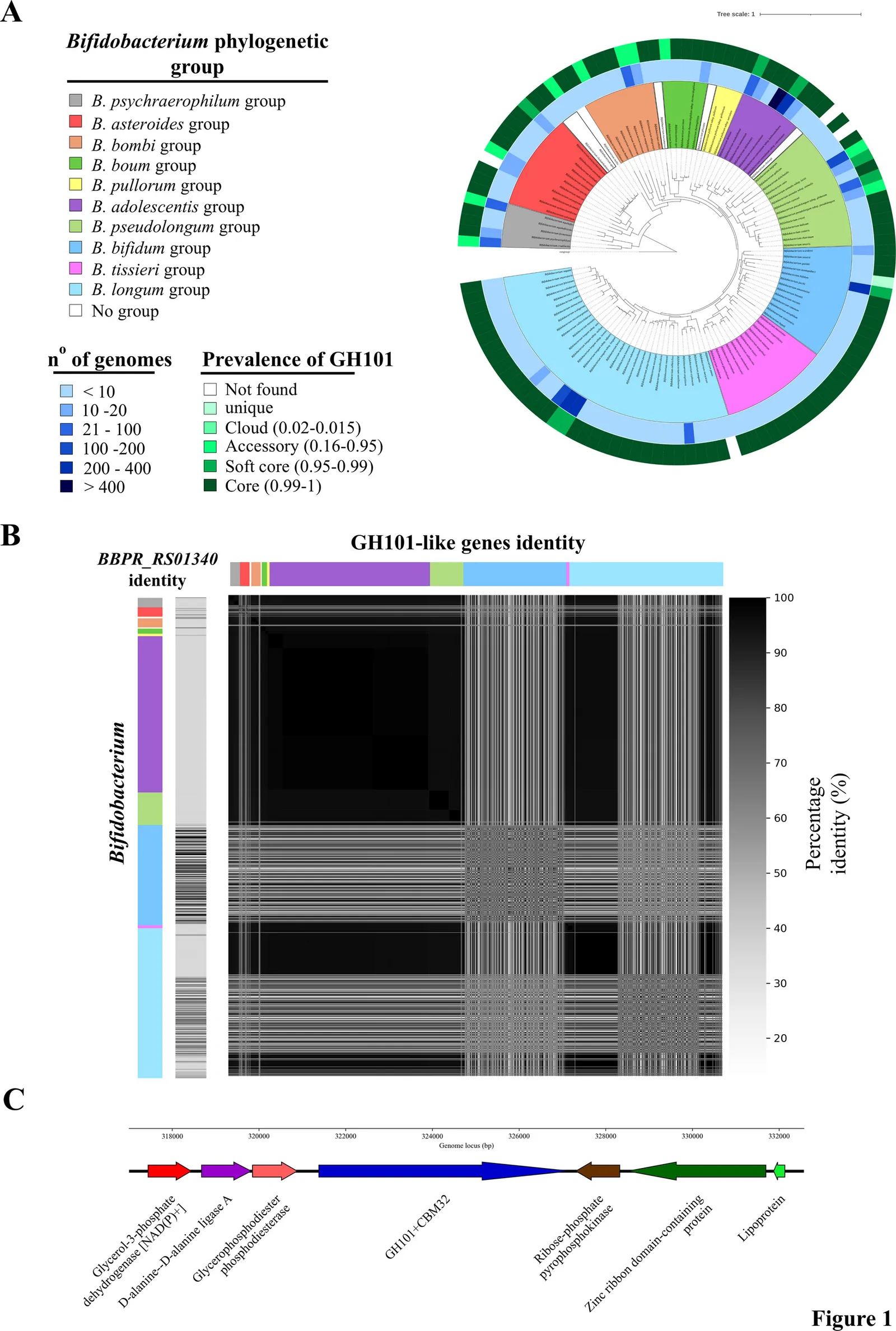
Phylogenetic distribution and sequence divergence of GH101 family enzymes within the *Bifidobacterium* genus. **A** Maximum-likelihood genomic tree based on the core genome of 117 bifidobacterial reference strains. The outer rings display the prevalence and distribution of the GH101 family among 2665 *Bifidobacterium* RefSeq NCBI genomes. Each bifidobacterial species has been highlighted in a different color according to its phylogenetic group. **B** Heatmap of the protein sequence identity matrix based on identified GH101-like COGs in bifidobacteria. Based on functional annotation and domain architecture, two clusters emerge: a highly conserved GH101 family, broadly distributed across the genus, and a more divergent multidomain GH101 + CBM32 variant, restricted to genomes belonging to the *B. bifidum* and *B. longum* phylogenetic groups. **C** Schematic representation of the genomic region surrounding BBPR_RS01340, showing the organization and transcriptional orientation of neighboring genes. Arrows indicate the direction of transcription, and the position of the insertion used to generate the mutant is indicated

To predict genes with GH101-like catalytic domains within the *Bifidobacterium* genus, the sequence of 2,665 RefSeq NCBI genomes encompassing all 117 bifidobacterial (sub)species was investigated (Lugli et al. 2021a, b;) (Supplementary Table S1). The reconstruction of the *Bifidobacterium* pangenome enabled an in-depth analysis of the genome functional annotations, revealing that 113 (sub)species harbored genes with a GH101-like domain (Fig. 1A), while *B. coryneforme*, *B. magnum*, *B. dolichotidis,* and *B. panos* were the only species lacking genes encoding GH101-like domains. Following the phylogenetic reconstruction of the genus, three distinct clusters of GH101-like orthologous genes (COGs) were identified among bifidobacteria (Fig. 1B). The first COG was found to be highly conserved across the genus, being present in 97% of extant species, and annotated as a “putative propionyl-CoA carboxylase beta chain 5” with a putative GH101 domain. Gene sequences of this COG have an average identity of 39.7 ± 0.5% to the BBPR_RS01340 gene sequence, which was previously predicted to encode a GH101 enzyme involved in the persistence of *B. bifidum* PRL2010 (Tarracchini et al. 2023). The second COG was identified in 20 bifidobacterial species across six phylogenetic groups of the genus, with an average identity of 60.6 ± 6.5% to BBPR_RS01340. The third COG included the closest homologs of the BBPR_RS01340 gene, with an average sequence identity of 88.9 ± 10.9% (99.1 ± 2% within the *B. bifidum* species and 77.5 ± 0.4% among genes from other species). Representatives of this COG were found in only nine species, i.e., *B. aerophilum*, *B. bifidum*, *B. leontopitheci*, and *B. samirii* of the *B. bifidum* phylogenetic group, and *B. colobi*, *B. imperatoris*, *B. longum*, *B. reuteri*, and *B. sanguini* of the *B. longum* phylogenetic group. More interestingly, genes belonging to this COG encode a GH101 catalytic domain coupled with a Carbohydrate-Binding Module 32 (CBM32) domain, unlike genes belonging to the other two COGs, which contain only a GH101-like domain. Accordingly, proteins with the CBM32 domain are known to recognize short mono- or oligosaccharides and non-reducing terminal carbohydrates, as well as promote efficient substrate capture, facilitating microbial utilization and uptake of the recognized carbohydrate (Abbott et al. 2007; Ficko-Blean & Boraston 2006; Newstead et al. 2005).

Results of the in silico analyses revealed that the GH101 domain is highly prevalent across the *Bifidobacterium* genus, with three COGs predicted to exhibit GH101 activity. BBPR_RS01340 homologs belong to a specific COG identified in only two phylogenetic groups, in which the GH101 domain is combined with a CBM32 domain. The gene BBPR_RS01340 displays a percentage identity of 66.53% against the homologous gene in *B. longum* EngBF (GenBank: AAX44931.1), in which GH101 was first described, with a sequence coverage of 98%. The presence of both domains may confer an evolutionary advantage to species colonizing and persisting in environments rich in complex carbohydrates with non-reducing ends, such as the human intestine, where the CBM32 activity may enhance the affinity and catalytic efficiency of GH101 for complex sugars on the surface of intestinal epithelial cells, including host glycans.

### Preparation of the B. bifidum GH101::pFREM30 mutant and evaluation of its stability

As previously reported, *B. bifidum* exhibits strong resistance to genetic transformation due to its robust cell wall and active restriction–modification (R/M) systems (Serafini et al. 2012). To address this limitation, a high-efficiency transformation protocol was optimized using the integration vector pFREM30, resulting in the mutant strain *B. bifidum* GH101::pFREM30. The stability of the mutant strain was assessed over 14 days using a plasmid stability assay (Figure S1). Subsequently, to further characterize the strain and to exclude any antibiotic effect, we investigated the ability of *B. bifidum* GH101::pFREM30 to retain the plasmid under sub-selective antibiotic conditions. This growth was achieved by performing assays across a range of chloramphenicol (CHL) concentrations, from 5 µg/mL down to 0.1 µg/mL. The results demonstrated that the mutant strain maintained the plasmid at all tested antibiotic concentrations (Figure S2). Accordingly, all subsequent experiments were conducted using the lowest CHL concentration to minimize potential effects associated with antibiotic exposure.

### Adhesion to mucus-secreting intestinal cells in Bifidobacterium bifidum PRL2010 GH101-deficient mutant

**Fig. 2:**
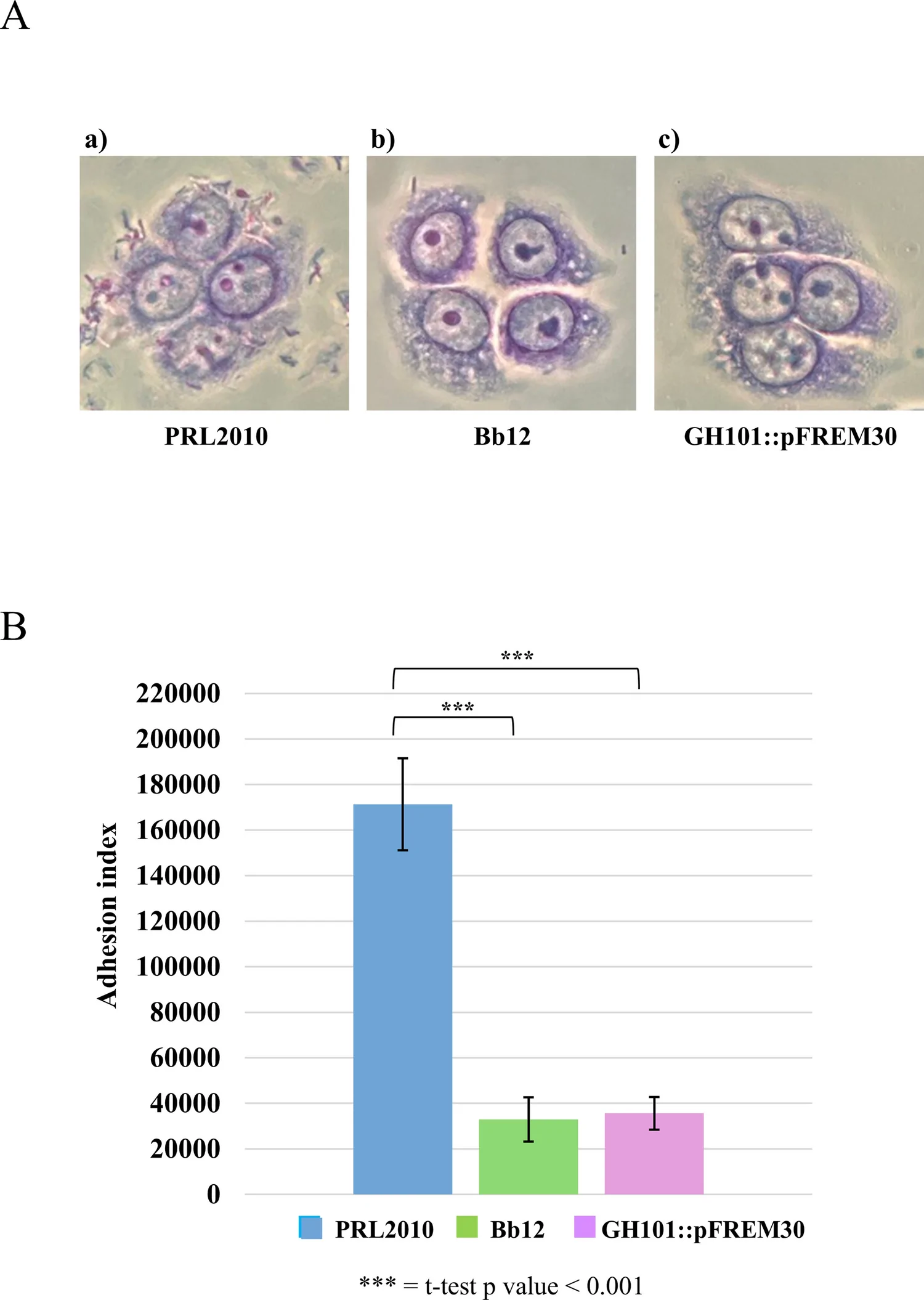
Adhesion to mucus-secreting intestinal cells in *Bifidobacterium bifidum* PRL2010 GH101-deficient mutant. **A** Optical microscopy images showing adhesion of *Bifidobacterium bifidum* PRL2010 (a), negative control *B. animalis* subsp. *lactis* Bb12 (b), and GH101-deficient mutant (c) to human mucus-secreting intestinal epithelial cells HT29-MTX. Bacteria appear as slender violet-stained bacilli adhering to the cell surface. Host cell nuclei are stained red/magenta. The images highlight the physical interaction between bacteria and host mucosal cells. **B** The quantification of the ability of *B. bifidum* PRL2010, *B. animalis* subsp. *lactis* Bb12, and the mutant strain to adhere to HT29-MTX secreting mucin cells. The error bars indicate standard error of the mean (SEM); the three asterisks indicate a *p*-value < 0.001 (*t*-test)

To confirm the contribution of the BBPR_RS01340 gene to *B. bifidum* PRL2010 persistence in the human gut via mucin anchoring, the adhesion of *B. bifidum* PRL2010 and *B. bifidum* GH101::pFREM30 to human mucin-secreting HT29-MTX cells was investigated. Adhesion assays on HT29-MTX cells showed significantly reduced attachment of the *B. bifidum* GH101::pFREM30 mutant compared to PRL2010 wild-type (*p* < 0.001) (Fig. 2A, B). Specifically, while the wild-type exhibited an adhesion index of approximately 171,333 ± 20,201, the mutant displayed a substantially lower value of 35,667 ± 7166 (Fig. 2B). Notably, the adhesion levels of the mutant were comparable to those of the *B. animalis* subsp. *lactis* BB12 reference strain (Argentini et al. 2024; Lugli et al. 2024), further supporting a substantial impairment in host cell interaction upon GH101 inactivation (Fig. 2B). Partial retention of adhesion in the mutant suggests functional redundancy within the glycoside hydrolase repertoire, with other enzymes partially compensating for the loss of GH101. The reduced adhesion likely reflects the mutant’s reduced ability to colonize and persist in the host’s gut, suggesting that GH101 is involved in the complex mucin metabolism of the species *Bifidobacterium bifidum*.

### Mucin O-glycans utilization of B. bifidum PRL2010 GH101-deficient mutant

**Fig. 3:**
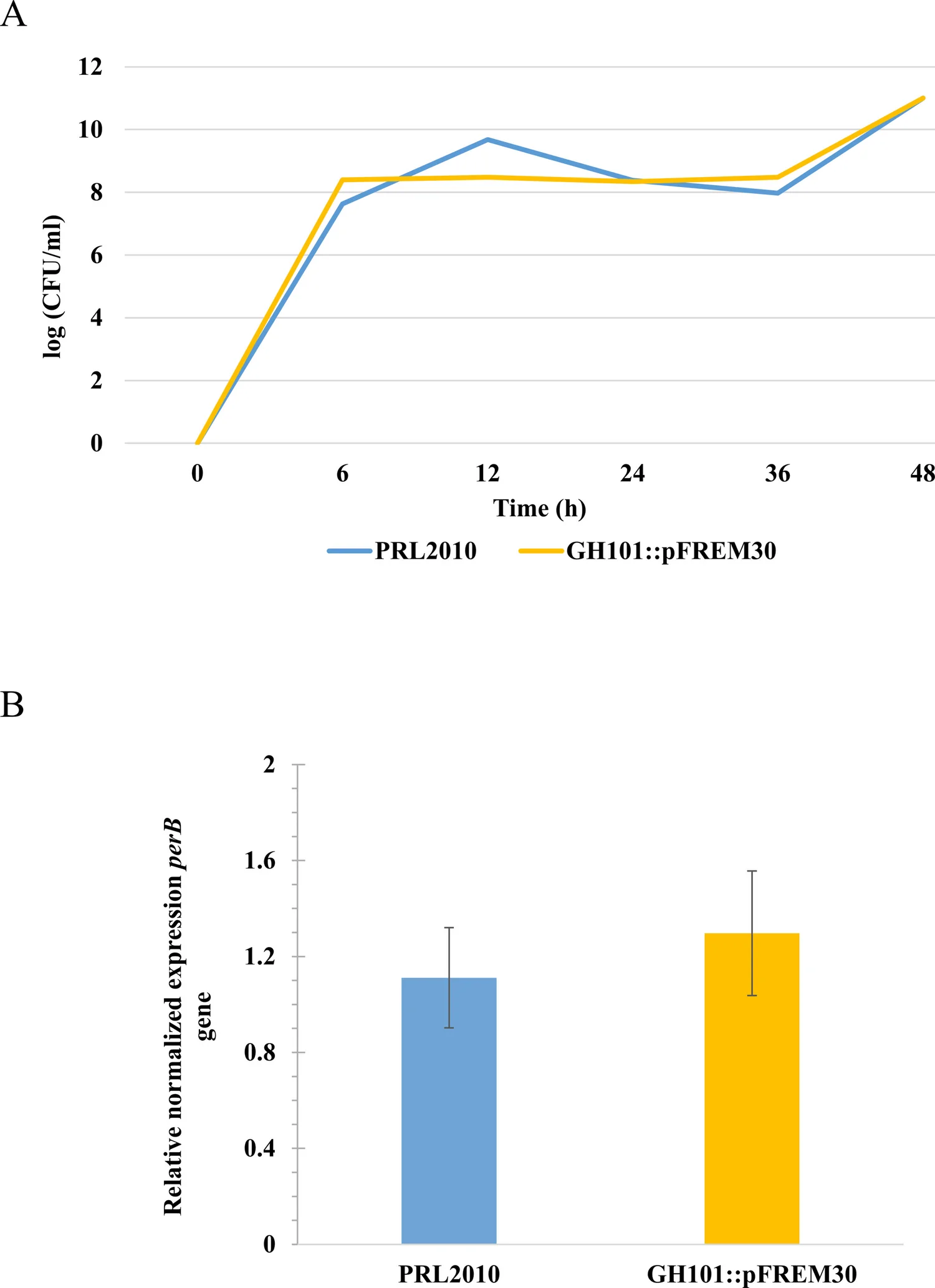
Mucin growth assay. **A** The growth profiles of *B. bifidum* PRL2010 and *B. bifidum* GH101::pFREM30, cultured in the presence of the minimum CHL concentration using mucin as the only carbon source. Bacterial growth was evaluated at 6, 12, 24, 36, and 48 h by viable cell plating. The x-axis indicates the sampling times, whereas the y-axis represents the logarithmic values of the total viable bacterial counts, reported as CFU/mL for each collected aliquot. The experiments were carried out in triplicate, and the data shown represent the mean values at each time point. **B** The relative transcription level of the *perB* gene that was differentially expressed in the wild-type and mutant strain by qPCR. The y-axis represents the normalized expression level (ΔCt) as calculated by the CFX96 Bio-Rad software. Values are shown as mean ± SD

The capacity of the *B. bifidum* GH101::pFREM30 mutant to utilize mucin as the sole carbon source was evaluated in comparison to the wild strain. Specifically, *B. bifidum* PRL2010 wild-type and the GH101-deficient mutant were cultivated in mMRS medium as a control, and in mMRS w/o carbon source and supplemented with 0.5% mucin. Growth of each strain was monitored at 0, 6, 12, 24, 36, and 48 h by colony-forming unit (CFU) enumeration on mMRS agar. Notably, the mutant exhibited no difference in growth performance in mucin-supplemented medium compared to the wild-type strain (Fig. 3A). This observation reflects the complexity of mucin as a substrate, which can be degraded by multiple enzymes, and suggests that other glycoside hydrolases encoded by the mutant PRL2010 strain (Turroni et al. 2010a, b; Tarracchini et al. 2023; Rizzo et al. 2024) may compensate for the absence of the functionality of the BBPR_RS01340 gene product. To test this hypothesis, we evaluated *perB* gene expression, which was previously identified as a major contributor to mucin degradation in *B. bifidum* PRL2010, encoding for a GH136 (Rizzo et al. 2024; Tarracchini et al. 2023). This analysis revealed that *perB* expression levels in the GH101::pFREM30 mutant were comparable (and slightly higher, though not statistically significant) to those observed in the wild-type strain when both strains were grown in a medium containing mucin as the sole carbon source (Fig. 3B). These findings supported the notion that the GH101::pFREM30 mutant's ability to grow similarly to the wild-type under these conditions is likely due to compensatory activity mediated by *perB*, which offsets the loss of GH101 function.

**Fig. 4:**
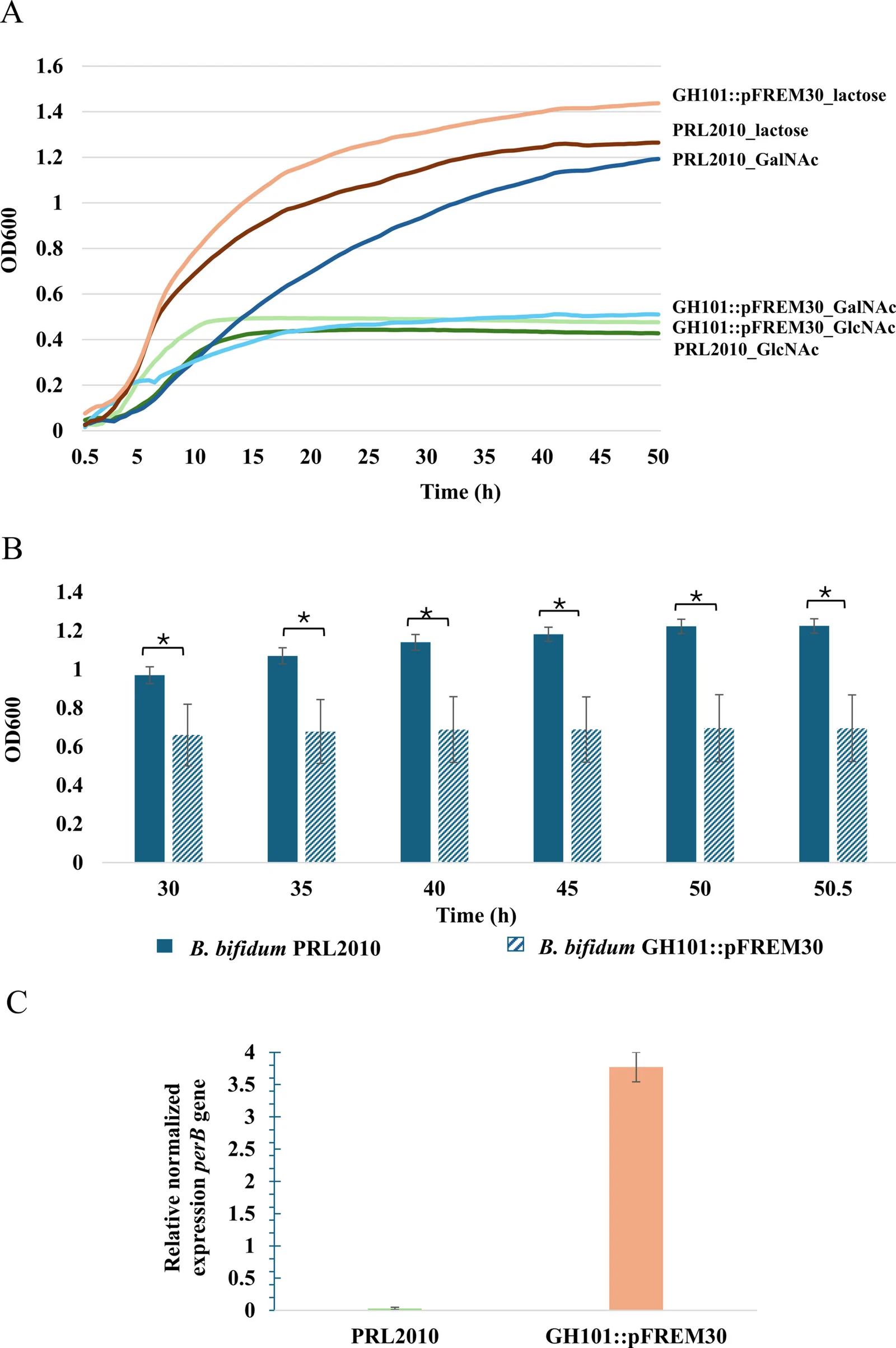
Evaluation of *Bifidobacterium bifidum* PRL2010 and *B. bifidum* GH101::pFREM30 ability to grow on O-GalNAc glycans residues. **A** Growth curves of *B. bifidum* PRL2010 and *B. bifidum* GH101::pFREM30 with different carbohydrates as the sole carbon source: Lactose, N-acetyl-galactosamine (GalNAc), and N-acetyl-glucosamine (GlcNAc). Growth was measured at OD600. Data represent means of three biological replicates analyzed using the Cerillo Labrador system. **B** shows the growth of *B. bifidum* PRL2010 and *B. bifidum* GH101::pFREM30 with GalNAc as the sole carbon source, at different time points. Each bar plot represents the average of the biological triplicate, and the horizontal bars indicate standard deviations. Statistically significant differences between strains are indicated by * (*p* < 0.05). **C** The relative transcription level of the *perB* gene that was differentially expressed in the wild-type and mutant strain by qPCR. Both strains were grown in MRS medium without glucose, with GalNAc added as the sole carbon source. The y-axis represents the normalized expression level (ΔCt) as calculated by the CFX96 Bio-Rad software. Values are shown as mean ± SD of three biological replicates

Since mucin O-GalNAc glycans are covalently α-linked via an N-acetylgalactosamine (GalNAc) moiety to serine or threonine by an O-glycosidic bond (Tailford et al. 2015a, b), we performed growth assays in mMRS without glucose supplemented with 1% N-acetyl-galactosamine (GalNAc) or 1% N-acetyl-glucosamine (GlcNAc). *B. bifidum* PRL2010 wild-type displayed distinct growth profiles, consistent with previous observations (Turroni et al. 2010a, b). In contrast, *B. bifidum* GH101::pFREM30 mutant exhibited reduced growth compared to the wild-type in both GalNAc and GlcNAc, except when grown on lactose (Fig. 4A). This exception likely reflects the relative stability of the lactose-containing medium for the mutant strain (Rizzo et al. 2024). Overall, the reduced growth of the mutant strain relative to the wild-type suggests that the gene BBPR_RS01340 is involved in the cleavage of N-acetyl-galactosamine from mucin. Moreover, a closer examination of growth differences between the two strains revealed that they became statistically significant (*p* < 0.05) at 30 h of incubation and remained significant throughout the experiment (Fig. 4B).

The phenotype observed is not directly explained by the known enzymatic activity of GH101 (EngBF), which targets Galβ1-3GalNAc structures in mucin O-glycans. A possible explanation is that the mutation of *engBF* function may have pleiotropic effects on the regulation of carbohydrate utilization systems. In particular, impaired release of GalNAc-containing oligosaccharides from mucin could indirectly influence the GalNAc uptake and/or catabolism, such as ABC transporters or specific metabolic enzymes. This regulatory response could reflect a substrate-dependent metabolic adaptation, where the inability to access complex O-glycans alters the cellular demand and regulation of free monosaccharide transport systems.

To further substantiate this observation, we investigated *perB* gene expression in both wild-type and mutant strains growing on GalNAc as the sole carbon source. This analysis revealed that, under this condition, the mutant *B. bifidum* GH101::pFREM30 exhibited a statistically significant upregulation of the GH136 gene compared to the wild-type strain. These findings further support the existence of a compensatory mechanism between glycoside hydrolases, whereby GH136 overexpression offsets the loss of GH101 activity (Fig. 4C).

## Discussion

Collectively, these findings support the hypothesis that GH101 from *B. bifidum* PRL2010 acts as a gatekeeper enzyme linking mucin degradation to both metabolic adaptation and effective host colonization. The enzyme-mediated access to mucin glycans directly influences bacterial adhesion and, consequently, may contribute to bifidobacterial persistence within the gut environment, reinforcing the concept that glycan foraging and mucosal interactions are tightly interconnected. Overall, our results identify GH101 as a key determinant of ecological fitness and host–microbe interactions in the human gut (Turroni et al. 2010a, b; Turroni et al. 2013).

This study establishes GH101 as a critical adaptive trait enabling *B. bifidum* PRL2010 to persist within and colonize the human gut mucosal niche. By initiating the degradation of host mucin O-glycans, GH101 provides access to host-derived carbohydrates, sustaining bacterial growth while simultaneously promoting close association with the mucus layer (Ruas-Madiedo et al. 2008; Egan et al. 2014; Tailford et al. 2015a, b; González-Morelo et al. 2020). A mutant of the BBPR_RS01340 gene disrupts these processes, leading to a marked reduction in mucosal adhesion and, consequently, impairing bacterial fitness in this important intestinal habitat. Our findings support a model in which efficient mucin degradation represents not only a metabolic advantage but also a prerequisite for stable colonization of the gut epithelium. Although partial functional compensation by other mucin-degrading enzymes, such as GH136, may occur under low-stress conditions, GH101 appears to play a non-redundant role in maintaining colonization efficiency, particularly under conditions that challenge bacterial survival.

We suggest that loss of GH101 activity may not only affect mucin glycan degradation but also indirectly modulate the uptake of GalNAc-specific transport and metabolic pathways, potentially explaining the selective impact observed on GalNAc utilization, while GlcNAc metabolism remains unaffected.

More broadly, these results highlight how specialization in host glycan foraging drives ecological adaptation within the gut microbiota (Bell & Juge 2021; Derrien et al. 2022). The association between GH101 expression and host-dependent persistence patterns further suggests that the enzymatic capacity for mucin degradation contributes to long-term strain stability and may underlie inter-individual and sex-dependent differences in microbiota composition. By linking a defined enzymatic function to mucosal adaptation and colonization, this work provides mechanistic insight into how *Bifidobacterium bifidum* establishes durable host associations and underscores the importance of host-derived glycans as key ecological drivers shaping microbiota structure and persistence (Koropatkin et al. 2012).

## Supplementary Information

Below is the link to the electronic supplementary material.

ESM 1 (PDF 2.33 MB)

ESM 2 (JPG 83.0 KB)

ESM 3 (XLSX 114 KB)

ESM 4 (JPG 53.4 KB)

ESM 5 (JPG 60.6 KB)

## Data Availability

No datasets were generated or analyzed during the current study.
