# Supplementary material for: Mucin O-glycan degradation by GH101 underpins mucosal persistence of Bifidobacterium bifidum PRL2010
Source: Appl Microbiol Biotechnol. 2026 Jul 18;110:287. doi: 10.1007/s00253-026-13964-1 (PMC13612775; doi:10.1007/s00253-026-13964-1)
Supplement: Supplementary file 1 [file 253_2026_13964_MOESM1_ESM.pdf]

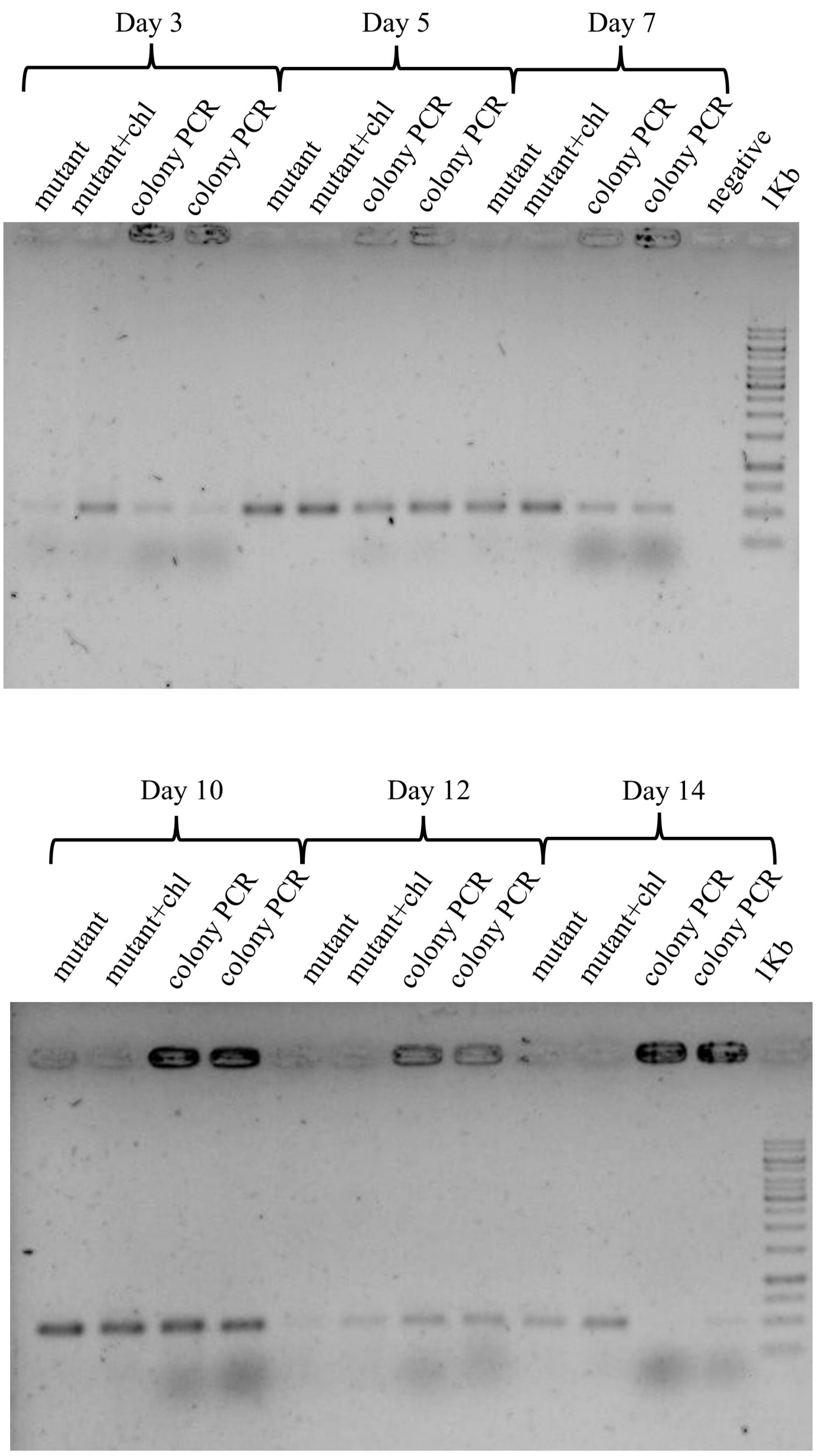

Figure S1: Mutant stability assay. Agarose gel electrophoresis (1.5% agarose) of PCR amplified products using species-specific PCR primer sets.

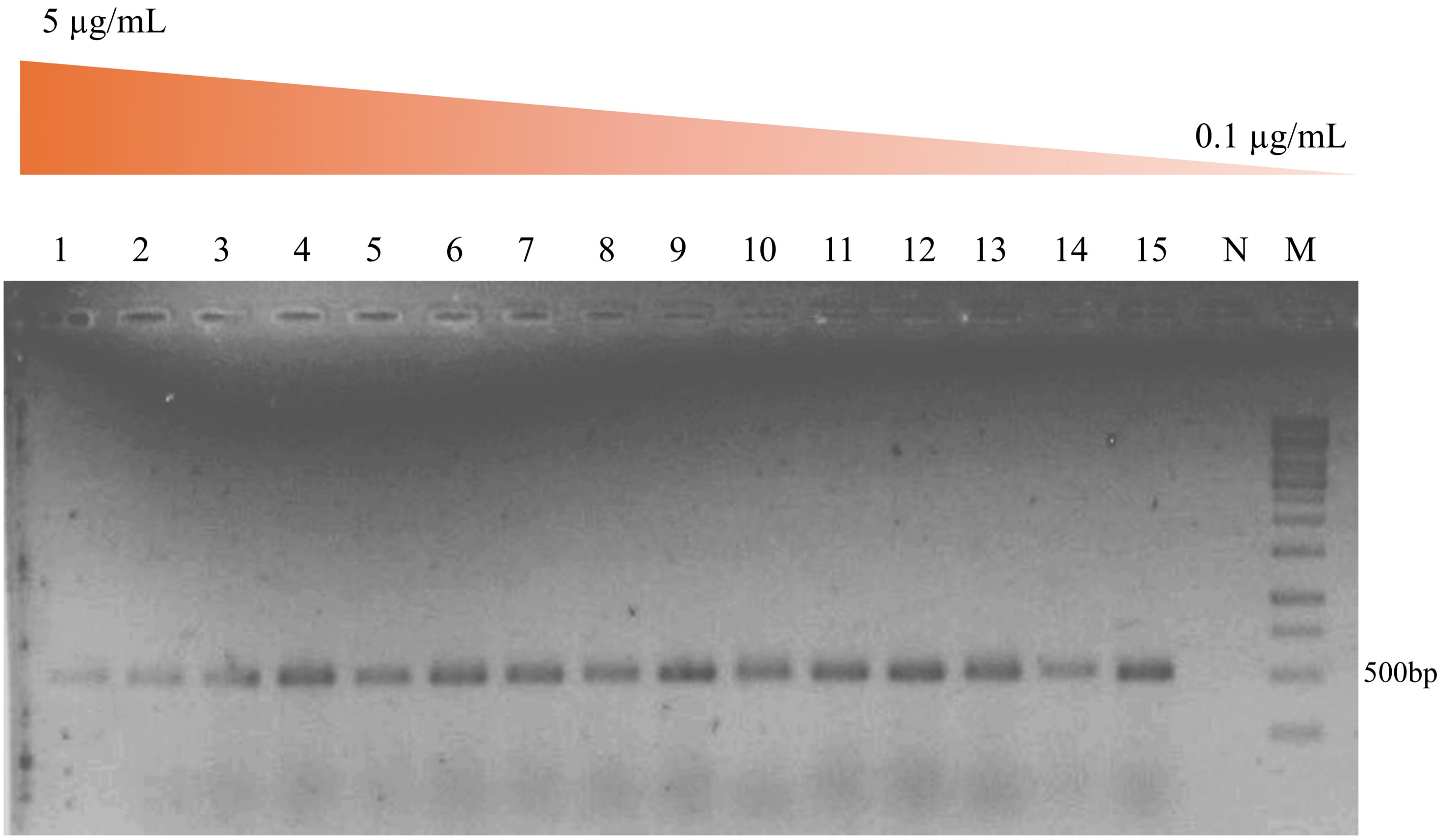

Figure S2: Plasmid maintenance assay under sub-selective conditions. Agarose gel electrophoresis (1.5%) of PCR products obtained from plasmid amplification after growth with different concentration of chloramphenicol (CHL), from 5µg/mL to 0.1 µg/mL. Lanes 1–3: amplified fragments with CHL 5µg/mL; lanes 4–6: amplified fragments 2µg/mL; lanes 7–9: amplified fragments 1µg/mL; lanes 10–12: amplified fragments 0.5 µg/mL; lanes 13–15: amplified fragments 0.1 µg/mL; lane N: negative control; lane M: DNA ladder (1 kb). The presence of a single band at ~ 500 bp confirms successful amplification of the target gene.

Table S2: RT-qPCR primers

	Gene	Primer	Sequence (5'->3')
housekeeping genes	*atpD*	Forward primer	TCGAAACCGGTATCAAGGTC
		Reverse primer	ACGTAGATGGCTTGCAGCGAGGTG
	rpoB	Forward primer	GGCAACAAGGGCGTCATCTC
		Reverse primer	CCAGCCGGCGTGNGCCACCC
	LDH	Forward primer	GGCGAGCACGGCGACTCCGA
		Reverse primer	CGNAGTTGGTWGCGCCTTG
	**BBPR_RS01340**	Forward primer	GTCACCAATGGCACGTTCAC
		Reverse primer	AGGCGTACTTGTTCACCTCG
